# Antimicrobial drug susceptibility testing for the management of *Helicobacter pylori* infection in personalized eradication therapy

**DOI:** 10.3389/fmicb.2025.1626930

**Published:** 2025-08-19

**Authors:** Lingzhu Gou, Zenghui Ma, Mengyu Han, Wenji Tian, Xinglan Chen, Xiaojuan Kang, Dekui Zhang

**Affiliations:** ^1^Department of Gastroenterology, Lanzhou University Second Hospital, Lanzhou, China; ^2^Department of Gastroenterology, Key Laboratory of Digestive Diseases of Lanzhou University Second Hospital, Lanzhou, China; ^3^Department of Respiratory, Gansu Second People’s Hospital, Lanzhou, China; ^4^The First People’s Hospital of Jiande City, Hangzhou, China; ^5^Department of Oncology, Gansu Provincial Central Hospital, Lanzhou, China

**Keywords:** *Helicobacter pylori*, antibiotics resistance, eradication treatment, culture-based methods, molecular-based methods

## Abstract

*Helicobacter pylori* is a gram-negative bacterium that associated with diseases such as gastritis, peptic ulcer and gastric cancer. In recent years, various treatment options have been evaluated, such as bismuth-containing quadruple therapy, high-dose dual therapy, and the use of acid-suppressing drugs such as Vonoprazan, however, the effectiveness of *H. pylori* eradication treatment is still dramatically decreasing due to the rising antibiotic resistance rate, and successful eradication of *H. pylori* has become a major public health problem. Therefore a promising strategy against drug-resistant *H. pylori* is to individualize treatment based on the outcome of antibiotic resistance. This article reviews the antibiotic resistance situation in recent years in various regions. The advantages and disadvantages of novel antibiotic resistance detection methods are examined, and the therapeutic efficacy of individualized therapy under different detection methods is evaluated. Molecular methods have developed rapidly in recent years, and non-invasive methods can quickly and accurately determine the presence of drug resistance. Clinical application of antibiotic resistance test results to guide medication use needs to be used as early as possible. Customized therapies based on antibiotic drug sensitivity testing and individualized therapies guided by personal medication history can contribute to future therapeutic strategies.

## 1 Introduction

With drug resistance rates of *Helicobacter pylori* (*H. pylori*) climbing and eradication rates declining globally this year, new breakthroughs need to be found in the eradication treatment of *H. pylori*. Unfortunately, curing this infection remains challenging for clinicians, as there are no available empirical treatment regimens capable of eradicating the bacteria in all treated patients. The application of drug susceptibility testing not only enables precise treatment but also prevents antimicrobial drug resistance. The 2022 Chinese Guidelines for the Treatment of *Helicobacter pylori* Infections propose individualized treatment guided by bacterial culture and antimicrobial drug susceptibility testing for patients with refractory *H. pylori* infections when available (Zhou et al., 2022), but there is still a lack of definitive evidence to support individualized rather than empirical treatment. There are various detection methods for drug resistance testing, and those with high specificity and sensitivity are still being explored. Literature published was systematically identified in PubMed from 2018 to 2025 using the following search terms: (*Helicobacter pylori*) AND (antibiotic OR antimicrobial OR antimicrobial OR antibacterial OR anti-bacterial OR drug) AND (resistance OR resistant*). Each protein was searched individually to reduce the rate of missed detection. This article compares the existing methods for drug resistance testing with the aim of clarifying the direction for future resistance testing investigations.

## 2 Detection of *Helicobacter pylori* antibiotic resistance

There are two different approaches for testing antimicrobial resistance in *H. pylori*: cultures with antimicrobial susceptibility testing (AST) and molecular testing. The AST are phenotypic identification methods used to isolate *H. pylori* from gastroscopic biopsy specimens and determine antibiotic susceptibility by agar dilution, disk diffusion, broth microdilution, or E-test. Molecular-based methods primarily examine mutations for specific antimicrobials, such as 23S rRNA and gyrA genes. These methods are mainly based on polymerase chain reaction (PCR), real-time PCR (qPCR), and others (Table 1). Next-generation sequencing (NGS), a technology capable of massively parallel sequencing, is a powerful tool for examining mutations in multiple regions. In addition to gastric mucosal biopsies specimens, studies on the use of non-invasive samples such as gastric juices and stool have obtaining promising results.

**Table 1:** Molecular methods to characterize antibiotic resistance in *H. pylori.*

Methods	Authors (year)	Sample	Antibiotic	Mutation site
PCR	Örsten et al. (2023)	Gastric mucosa biopsy specimen	Clarithromycin	A2143G, A2142G, A1983G, and A2142C
			Amoxicillin	T2188C, T2182C, G1949A, and A2142G
PCR	Ismail et al. (2023)	Gastric mucosa biopsy specimen	Clarithromycin	A2143G and D59N+
			Metronidazole	N87I
			Levofloxacin	D91G
PCR	Nguyen et al. (2023)	Gastric mucosa biopsy specimen	Clarithromycin	A2143G
Nested PCR	Karmakar et al. (2023)	Gastric mucosa biopsy specimen	Clarithromycin	A2143G
qPCR	Kocazeybek et al. (2019)	Gastric mucosa biopsy specimen	Clarithromycin	A2115G, G2141A, and A2144T
qPCR	Contreras et al. (2019)	Gastric mucosa biopsy specimen	Tetracycline	AGA(926)GGA
qPCR	Tran et al. (2022)	Gastric mucosa biopsy specimen	Clarithromycin	Phe366Leu, Ser414Arg, Glu/Asn464-466, Val469Met, Phe473Val, and Asp479Glu
qPCR	Burayzat et al. (2023)	Gastric mucosa biopsy specimen	Clarithromycin	A2142G and A2143G
qPCR	Noh et al. (2023)	Gastric mucosa biopsy specimen	Clarithromycin	A2143G and A2142G
qPCR	Alarcón-Millán et al. (2023)	Gastric mucosa biopsy specimen	Clarithromycin	A2143G and A2142C
In-house qPCR	Hays et al. (2019)	Gastric mucosa biopsy specimen	Clarithromycin	A2142G, A2142C, A2143G, A2142T, +S
Amplidiag assay				A2142C, A2142G, A2143G, +S
PNA-qPCR	Nahm et al. (2019)	Gastric mucosa biopsy specimen	Clarithromycin	A2142G/A2143G
*H. pylori* molecular POCT kit	Tsuda et al. (2022)	Gastric juice	Clarithromycin	2,142 and 2,143
multiplex qPCR	Binmaeil et al. (2021)	Gastric mucosa biopsy specimen	Clarithromycin	A2142G,+S
			Levofloxacin	N87K, D91N, D91Y, and N87I
multiplex qPCR	Mormeneo Bayo et al. (2023)	Gastric mucosa biopsy specimen	Clarithromycin	A2143G and A2142G
multiplex qPCR	Zhao et al. (2022)	Gastric mucosa biopsy specimen	Clarithromycin	A2143G
			Levofloxacin	G271A/T, A272G, and C261A/G
multiplex qPCR	Kim et al. (2023)	Gastric mucosa biopsy specimen	Clarithromycin	A2143G and A2142G
Allele-specific PCR	Albasha et al. (2021)	Gastric mucosa biopsy specimen	Clarithromycin	A2142G and A2143G
Allele-specific PCR	Hamidi et al. (2020)	Gastric mucosa biopsy specimen	Clarithromycin	A2142G, A2143G, and A2143G + S
ARMS-PCR	Zhang et al. (2019)	Gastric mucosa biopsy specimen	Clarithromycin	A2143G, A2142G, and A2142C
ARMS-PCR	Li et al. (2020)	Gastric mucosa biopsy specimen	Clarithromycin	2143G and 2142G
			Levofloxacin	261A, 261G, 272G, 271 T, 271A, 259 T, and 260 T
ARMS-PCR	Zhang et al. (2021)	Gastric mucosa biopsy specimen	Clarithromycin	A2143G, A2142G, and A2142C
			Levofloxacin	C261A, G271A, A272G, T261G, G271T, and A260T
PCR reverse dot blot	Dai et al. (2022)	Gastric mucosa biopsy specimen	Amoxicillin	C556G, A562T, and A562G
			Levofloxacin	T87A, A91G, G91T, and G91A
			Tetracycline	926–928 (AGA to TTC) and 926–927 (AG to GT)
			Clarithromycin	A2142G and A2143G
PCR reverse dot blot	Wang et al. (2021)	Gastric mucosa biopsy specimen	Amoxicillin	T556S, N562Y, and N562D
			Levofloxacin	N87K, D91G, D91Y, and D91N
			Metronidazole	G616A
			Clarithromycin	A2142G and A2143G
			Tetracycline	AGA926-928TTC and AG926-927GT
RFLP	Roldán et al. (2019)	Gastric mucosa biopsy specimen	Clarithromycin	A2142G and A2143G
RFLP	Hussein et al. (2022)	Gastric mucosa biopsy specimen	Clarithromycin	A2143G and A2144G
RAPD	Farzi et al. (2019)	Gastric mucosa biopsy specimen	Clarithromycin	A2142G, A2143G and A2142C
LAMP	Park et al. (2021)	Gastric mucosa biopsy specimen	Clarithromycin	A2143G and T2182C

### 2.1 Culture-based method

The culture method can be divided into broth microdilution, disk diffusion, agar dilution, and E-test. The Clinical and Laboratory Standards Institute (CLSI) recommends agar dilution as the “gold standard” (Shakir et al., 2022). This method requires multiple fresh blood agar plates, which is time-consuming and laborious. Therefore, it is less commonly used for routine antibiotic sensitivity testing of clinical isolates. Disk diffusion and broth microdilution are the two most commonly used methods for clinical pathogens. E-test is a simple method that can be utilized to determine the Minimum Inhibitory Concentration (MIC) value of a single clinical isolate by using different concentration gradients on a single strip of paper. The studies showed good agreement between clarithromycin and levofloxacin when compared to broth microdilution and agar dilution methods (Tang et al., 2022). E-test and agar dilution methods compared with levofloxacin, metronidazole, tetracycline and clarithromycin showed good agreement and amoxicillin correlation was poor (Miftahussurur et al., 2020). The disk diffusion method compared with E-test showed good agreement for levofloxacin, clarithromycin and metronidazole and poor correlation for amoxicillin and tetracycline (Tang et al., 2020). E-test can be used as an alternative to agar dilution as a common clinical test for *H. pylori* drug sensitivity.

### 2.2 Molecular-based methods

The mechanism of *H. pylori* resistance to key antibiotics is well understood. Clinical resistance is caused by mutations in the nucleotide and amino acid sequences of *H. pylori* at the binding site to antibiotics. The molecular method can detect allelic mutation sequence sites, which is easier, faster, and more successful than the culture method. Additionally, it can detect heterogeneous resistance (Hays et al., 2019). Molecular methods can examine mutations for clarithromycin and levofloxacin. Clarithromycin resistance is mainly due to the point mutations especially at the A2142 and A2143 of 23S rRNA. The mutation sites of fluoroquinolones leading to resistance are in positions 86, 87, 88, and 91 of the genes gyrA and gyrB in DNA gyrase (Egli et al., 2020). However, molecular methods currently have limitations in detecting resistance to tetracycline (16S rRNA), rifampicin (rpoB), and metronidazole. In recent years, novel methods for detecting resistance to *H. pylori* have emerged.

Real-time quantitative fluorescence PCR (qPCR) allows for the direct amplification and analysis of gastric mucosal specimens, including formalin-fixed, paraffin-embedded samples, without the requirement for incubation and agar gel electrophoresis. qPCR is now more widely used in clinical practice because it enables easy and accurate interpretation of mutations directly through the signal of fluorescence melting peaks. Several modified qPCR methods and commercial kits have been developed.

A peptide nucleotide acid (PNA) probe-based qPCR test is a method with a PNA probe that allows for perfect hybridization, partial hybridization, and mismatch hybridization with template alleles. The genotypes of the alleles were obtained by analyzing the quenching temperature. PNA qPCR and dual priming oligonucleotide (DPO)-based multiplex PCR were used to analyze the A142G and A2143G mutation types, and the results were compared with those obtained using conventional PCR. The sensitivity and specificity of PNA qPCR were 100 and 100%, whereas the sensitivity and specificity of DPO-based PCR were 92.9 and 100%. DPO-based PCR involves two steps, PCR and agarose gel electrophoresis, whereas PNA qPCR only requires a single step of the real-time fluorescence PCR instrument, making it simpler and more accurate (Bénéjat et al., 2021).

The ready-to-use PCR microtiter plate is based on the principle of qPCR with optimized premix and emitter probe fluorophores to detect mutations at clarithromycin A2142G, A2143G, and A2142C sites. The time consumption, sensitivity, and specificity of this test strip need further exploration (Nahm et al., 2019).

To simultaneously detect resistance mutation sites in clarithromycin and levofloxacin, the researchers developed a multiplexed qPCR assay for the detection of mutations in clarithromycin 23S rRNA (A2142 and A2143) and levofloxacin gyrA (N87K and N87I, as well as D91N and D91Y). qPCR targeting the 23S rRNA showed a sensitivity of 100% and a specificity of 98.7%, while that targeting the gyrA gene demonstrated a sensitivity of 100% and a specificity of 99.8%. The method has high performance in detecting resistance to levofloxacin and is in good agreement with the E-test (Binmaeil et al., 2021).

Allplex™ is a quantitative multiplex PCR for the detection of clarithromycin A2142G, A2143G, and A2142C mutation sites. It is more sensitive (97.6% vs. 88.3%) and specific (96.0% vs. 82.0%) than DPO-PCR. Allplex™ showed higher accuracy in detecting the A2142G mutation site, which has a lower mutation rate (Kim et al., 2023).

The amount of *H. pylori* DNA template extracted from gastric mucosal specimens was small. To enhance the detection rate and quickly determine drug resistance directly from biopsy samples, a nested-allele specific primer-polymerase chain reaction (nested-ASP-PCR) was developed. Nested-ASP-PCR involves two steps. The first step includes amplifying 23S rRNA using DNA templates from biopsy samples. In the second step, the amplification product from the first step is used as a template and amplified using allele-specific primers for clarithromycin -sensitive or drug-resistant phenotypes. Results were in high concordance with Sanger sequencing. Nested-ASP-PCR only detects the A2143G mutation and not any other single nucleotide polymorphisms (SNPs) on the 23S rRNA. Multi-step PCR can be contaminated, leading to a high rate of false positives (Karmakar et al., 2023).

The amplification refractory mutation system combined with quantitative real-time PCR (ARMS-PCR) is based on designing specific ARMS primers that target mismatches at the 3′ end of the main chain, enhancing specificity and sensitivity. The ARMS-PCR method involves only requires 2 steps: nucleic acid extraction from gastric mucosal specimens and PCR amplification, which can be completed within 2 h. APMS-PCR has higher sensitivity than sequencing when the mutation rate is low. High concordance with the E-test method (97.1%, *p* > 0.05) was observed. ARMS-PCR requires optimization of the primers and fluorescent probes to target as many different sites as possible to prevent a decrease in the detection rate (Zhang et al., 2019). Multiplex ARMS-PCR in a single tube enables the detection of clarithromycin and levofloxacin resistance mutation sites, optimized for simplicity and speed. Compared with Sanger sequencing, multiplex ARMS-PCR is highly sensitive and specific, especially for detecting clarithromycin (Li et al., 2020).

PCR-RLFP can detect point mutations at common mutation sites in clarithromycin. Different restriction endonucleases can cut different point mutations into DNA fragments of different sizes. The fragments can be identified by separating and digesting them on an agar gel. PCR-RFLP does not require sequencing and is highly cost-effective. Detection time can be shortened compared with culture method, but it is necessary to isolate gastric mucosal *H. pylori* strains to extract nucleic acids, which may appear to reduce the chance of detection due to culture difficulties. Some less common mutations cannot be detected by RFLP (Hussein et al., 2022).

Allele-specific PCR is the use of multiple pairs of primers to amplify strain DNA templates, where nucleotides with different point mutations will bind to different primers, yielding PCR products of different lengths. Allele-specific PCR extracts DNA templates directly from the gastric mucosa without the need for isolation and culture. Alleles can detect mixed infections (Hamidi et al., 2020). Allele-specific PCR targeted only two common mutations. Allele-specific PCR was less sensitive compared to sequencing (allele PCR results: 9/53 A2142G, no A2143G, sequencing results: 1/25A2142G, 5/25A2143G) (Albasha et al., 2021).

Loop-mediated isothermal amplification (LAMP) amplifies DNA at 60–65°C without the need for a thermal cycler, and DNA amplification can be visualized by dye staining after 30 min. Park CG et al. detected two mutation sites, 23S rRNA A2143G and T2182C mutation sites. DNA was extracted from biopsy tissues. LAMP can detect *H. pylori* infection and drug resistance mutation sites, and the results are in high concordance with RUT and PCR sequencing results (Park et al., 2021).

Hybridization, washing, and color development of the amplified product, the mutation point can be changed to blue color by naked eye (Dai et al., 2022). The time-consuming and accuracy of the method still needs to be further explored.

Due to increased sensitivity and specificity, non-invasive assays have recently been developed for fecal and gastric fluids, etc. Smart Gene™'s *Helicobacter pylori* Molecular Evening Test (POCT) kit detects the *H. pylori* Clarithromycin 23S rRNA mutations at positions 2,142 and 2,143 in gastric fluids. Nucleic acid extraction, amplification and detection are automated. The test can be completed in approximately 1 h. all mutations at position 2,142 can be detected by the POCT kit, but only mutations at position 2,143 with a mutation rate of 15% or higher can be detected. The sensitivity is 91.7% and the specificity is 100% compared to the broth dilution method (Tsuda et al., 2022). Pichon M et al. investigated *H. pylori* clarithromycin resistance in fecal samples using qPCR with a sensitivity and specificity of 96.3 and 98.7%, respectively (Pichon et al., 2022). Meta-analysis by Gong, R. J. et al. showed that PCR-based analysis of fecal samples had high diagnostic accuracy for detecting clarithromycin resistance in patients with *H. pylori* infection. The combined sensitivity was 0.91 (95% CI: 0.83 ~ 0.95) and the combined specificity was 0.97 (95% CI: 0.62 ~ 1.00) (Gong et al., 2021).

Compared with the culture method, most commercial systematic molecular methods are only capable of detecting two antibiotics, clarithromycin and levofloxacin, and a single specific mutation site in the target gene. Therefore, both methods are necessary for the study of drug resistance, and the mechanism of antibiotic resistance needs to be further explored, with a view to discovering assays with higher clinical utility.

## 3 Tailored therapy guided by antibiotic susceptibility testing

Pre-medication resistance testing can prevent resistance-related treatment failure and the emergence of resistance caused by antibiotic misuse, but due to the high cost and workload of the *H. pylori* culture method, molecular-based AST is an emerging method for obtaining *H. pylori* resistance through mutations in genes associated with resistance, but due to genetic diversity, the clinical efficacy of molecular-based AST for individualized treatment needs further study. Moreover, the eradication rate is related to the host’s own factors such as PPI-associated CYP2C19 polymorphisms, and some authors have suggested that empirical treatment based on patients’ medication histories and local resistance rates can be an alternative to pharmacovigilance-guided individualized testing (Ishibashi et al., 2023) (Table 2).

**Table 2:** Effectiveness of *H. pylori* drug resistance testing methods for detection and individualized treatment.

Methods		Sample	Analytical performance	Eradication rate
Culture-based Methods	Broth Microdilution	Gastric Biopsies	Coincidence Rate: Clarithromycin: 100%; Levofloxacin: 96%	–
	Disk Diffusion	Gastric Biopsies	Coincidence Rate: Metronidazole: 96.7%; Clarithromycin: 96%; Levofloxacin: 98.6%	–
	Agar Dilution	Gastric Biopsies	“Gold Standard”	First-line Treatment: ITT: 93.1%; PP: 100%. Salvage Treament: ITT: 78.10%; PP: 87.10%
	E-test	Gastric Biopsies	Coincidence Rate: Amoxicillin:93.1%; Metronidazole: 84.7%; Clarithromycin: 94.4%; Levofloxacin: 93.1%; Tetracycline:97.2%	First-line Treatment: ITT: 92.5%; PP: 100%
Molecular-based Methods	PCR + First-Generation Sequencing	Gastric Biopsies	–	Salvage Treament: ITT: 98%; PP: 98.4%
		Gastric Fluid	Clarithromycin: Sensitivity: 78%; Specificity: 100%. Levofloxacin: Sensitivity: 75%; Specificity: 99%	–
	qPCR	Gastric Biopsies	Clarithromycin: Sensitivity: 89.7%; Specificity: 100%. Levofloxacin: Sensitivity: 99.8%; Specificity: 100%	–
		stool	Sensitivity: 96.3%; Specificity: 98.7%	ITT: 87.44%; mITT: 89.23%; PP: 94.57%
	PNA probe-based qPCR test	Gastric Biopsies	Sensitivity: 100%; Specificity: 100%	–
	DPO-PCR	Gastric Biopsies	Sensitivity: 92.9%; Specificity: 100%	First-line Treatment: mITT: 92%; PP: 92.62%.
	Nested-ASP-PCR	Gastric Biopsies	Sensitivity: 100%; Specificity: 100%	–
	ARMS-PCR	Gastric Biopsies	Clarithromycin: Sensitivity: 100%; Specificity: 100%. Levofloxacin: Sensitivity: 98.04%; Specificity: 95.04%	–
	PCR-RLFP	Gastric Biopsies	–	–
	Allele-specific PCR	Gastric Biopsies	–	–
	LAMP	Gastric Biopsies	Coincidence Rate: 97.7%	–
	PCR reverse dot blot Preservation	Gastric Biopsies	–	–
	Smart Gene™	Gastric Fluid	Sensitivity: 91.7%; Specificity: 100%	–
	Allplex™	Gastric Biopsies	Clarithromycin: Sensitivity: 97.6%; Specificity: 96.0%	–
	Next-Generation Sequencing	Gastric Biopsies	–	91.70%

### 3.1 Personalized treatment guided by culture detection

In the first-line treatment, the individualized treatment group had a significantly higher eradication rate than empirical treatment and a lower incidence of adverse events (Perkovic et al., 2021). Meta-analysis by Francesco, V. et al. showed higher eradication rates with individualized treatment than with empirical treatment (89.7% vs. 77.6%). Individualized treatment prior to third-line therapy was significantly more effective than empirical treatment in achieving optimal eradication rates (>90%) (Francesco et al., 2022). For second-line and above-treated patients, AST and personal medication history-guided therapy (PMH) eradication rates were comparable (intention-to-treat analysis:78.10% vs. 74.29%, *p* = 0.42; protocol analysis:87.10% vs. 88.64%, *p* = 0.80), and the healthcare costs of pmh-guided therapy were lower (Ji et al., 2020).

### 3.2 Personalized treatment guided by molecular detection

In recent years, there have been numerous studies on individualized treatment for clarithromycin-related conditions using primer-based dual-start oligonucleotide primer (DPO) technology-based multiplex PCR. However, the results have been inconsistent, with variations in control group therapies and treatment durations. Most studies have administered a bismuth-containing quadruple therapy regimen without clarithromycin to patients with clarithromycin resistance in the individualized treatment group. While sensitive patients were given a three-drug regimen containing clarithromycin. In the empirical treatment group, different studies administered varying treatment regimens to patients (including three-drug regimens containing clarithromycin, three-drug regimens without clarithromycin, four-drug regimens containing clarithromycin, or four-drug regimens without clarithromycin), and the comparison of eradication rates across groups yielded inconsistent results. Compared with the clarithromycin-containing triple therapy, the eradication rate in the individualized therapy group was significantly higher than that in the empirical therapy group (Cho et al., 2019); compared with the clarithromycin-free triple therapy, even the eradication rate in the empirical therapy group was significantly higher than that in the individualized therapy group (Choi et al., 2021); compared with the clarithromycin-containing quadruple therapy, the eradication rate in the individualized therapy group was significantly higher than that in the empirical therapy group (Kim et al., 2022); when compared with the quadruple therapy regimen without clarithromycin but containing bismuth, there was no significant difference in eradication rates between the two groups (Cho et al., 2022). A recent study proposed a treatment regimen selection strategy: in empirical therapy, a combination of three antibiotics (clarithromycin, amoxicillin, and metronidazole) with a proton pump inhibitor (PPI), in individualized therapy, a regimen of clarithromycin, amoxicillin, and PPI for clarithromycin-sensitive patients, and a quadruple therapy regimen of oxytetracycline, metronidazole, PPI, and bismuth for clarithromycin-resistant patients. The results showed that the eradication rate in the individualized therapy group was significantly higher than that in the empirical therapy group, and adverse reactions were significantly lower than those in the empirical therapy group (Cho et al., 2025). All of the above detection methods are based on primer-double-start oligonucleotide primer (DPO technology) multiplex PCR to detect clarithromycin resistance by detecting mutations at the 23S RNA A2142G and A2143G sites. DPO-based molecular diagnosis may not only be used for the selection of sensitive antibiotics but also has guiding value in the application of bismuth-containing quadruple therapy.

Most published studies have used clarithromycin testing for first-line therapy. Choi et al. (2019) reported that in second-line therapy, the individualized treatment group (resistant group: PPI + tetracycline + metronidazole + bismuth compound, sensitive group: PPI + amoxicillin + clarithromycin) had a higher eradication rate than empirical therapy (PPI + tetracycline + metronidazole + bismuth compound) (90.1% vs. 96.0%, *p* < 0.001), and adverse reactions were significantly lower than those in empirical therapy. A randomized clinical trial in Taiwan evaluated the combined resistance of clarithromycin and levofloxacin in patients with refractory Hp infection. The results showed that individualized treatment based on resistance gene testing did not demonstrate a significant advantage over individualized treatment based on medication history (ITT analysis: 78% vs. 72.2%, *p* = 0.170; PP analysis: 78.4% vs. 74.4%, *p* = 0.346) (Liou et al., 2018). This cannot rule out the influence of factors such as the selection of treatment regimens and the effectiveness of conventional PCR testing, so further prospective studies are needed to determine the optimal salvage regimen for such patients.

New *H. pylori* resistance detection technologies developed in recent years, as mentioned earlier, have higher sensitivity and specificity than DPO PCR and conventional PCR, so it is necessary to explore the clinical value of these new technologies. Additionally, regarding the selection of resistant gene mutation sites, in a study by Cho et al. (2023) administering 500 mg of clarithromycin and extending the treatment duration to more than 7 days for patients negative for the A2142G and A2143G mutation sites did not improve the eradication rate. Although the common clarithromycin resistance mutation sites are A2142G and A2143G, we have reason to suspect that these two sites may not fully reflect Hp’s sensitivity to clarithromycin, and more sites are needed.

Due to the invasive nature of gastric mucosal samples and the difficulty in obtaining them, PCR techniques based on fecal samples have gained attention in recent years due to their non-invasive, convenient, rapid, and low-cost characteristics. Currently, PCR methods used for detecting fecal samples include nested PCR, real-time fluorescent quantitative PCR, and conventional PCR. A meta-analysis showed that molecular methods for detecting *H. pylori* resistance in feces have high sensitivity (0.97) and specificity (0.98) (Al Qady et al., 2025). In recent years, randomized controlled studies have been conducted to detect *H. pylori* resistance to clarithromycin and levofloxacin by identifying point mutations in the 23S rRNA and gyrA genes in fecal samples, with the aim of guiding clinical drug use based on resistance profiles. The results showed that compared to empirical treatment and individualized treatment regimens based on clarithromycin usage history, individualized treatment based on fecal resistance gene detection results achieved higher eradication rates (Yu et al., 2025). In addition to stool samples, gastric fluid is another non-invasive sample that can be obtained. Studies have shown that genetic testing of clarithromycin and levofloxacin from gastric aspirates has high accuracy rates of 97 and 95%, respectively (Vasapolli et al., 2025). However, there are currently no reported studies on the use of gastric fluid samples to guide clinical drug use.

### 3.3 Personalized treatment guided by NGS detection

Guo et al. (2022) detected *H. pylori* resistance to clarithromycin, levofloxacin, amoxicillin, and metronidazole in refractory patients using NGS and agar dilution, with a concordance rate higher than 90%, and eradication therapy was performed in this patient based on the results of the drug sensitivity test, and the eradication rates based on AST, PCR, and WGS were 90.9% (10/11), 83.3% (10/12), and 91.7% (11/12), which suggests that genotypic resistance-guided therapy may achieve satisfactory results with phenotypic resistance-guided therapy.

### 3.4 Comparison of genotype- and phenotype-guided individualized treatment

Chen et al. (2023) tested for resistance to clarithromycin and levofloxacin using PCR direct sequencing and agarose dilution methods in patients on first- and third-line treatment, respectively, and gave different eradication regimens based on the results. The results showed that molecular assay-guided therapy in first-line treatment had similar results to culture assay-guided therapy (eradication rate: 86% vs. 87%, *p* = 0.81, rate of difference: −0.7%), and molecular assay-guided therapy in third-line treatment was not inferior to culture assay-guided therapy results (eradication rate: 88% vs. 87%, *p* = 0.74, rate of difference: 1.3%).

## 4 Conclusion

**Figure 1:**
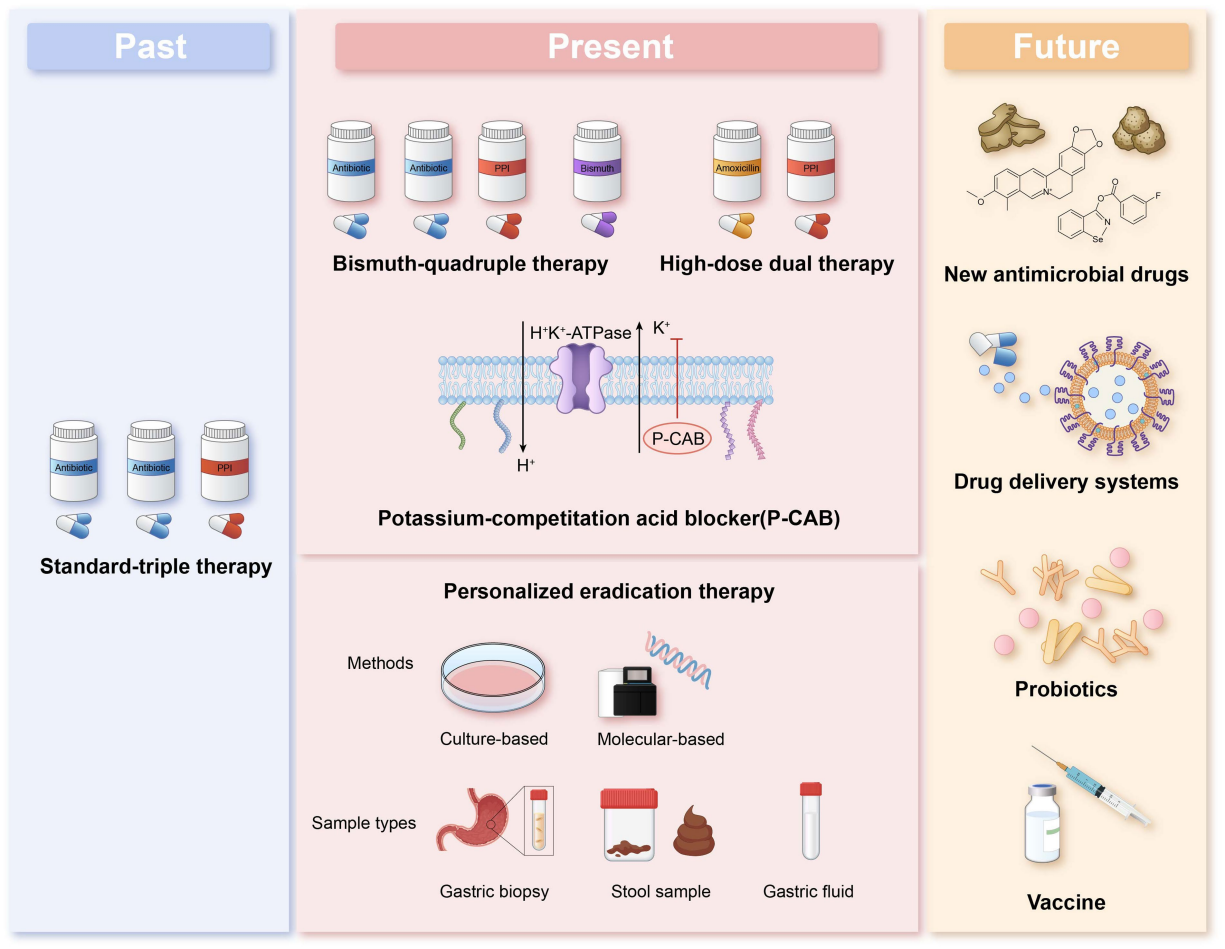
The past, present and future of *H. pylori* management.

This article reviews the current status of *Helicobacter pylori* antibiotic resistance, commonly used resistance detection methods, and the efficacy of eradication therapy guided by different methods. It is worth noting that the current situation of antibiotic resistance in *Helicobacter pylori* remains severe. Non-invasive molecular detection methods show significant potential for future development. In the future, empirical treatment should be replaced by individualized treatment. However, there is currently limited clinical evidence to suggest that individualized treatment guided by antibiotic susceptibility testing is superior to treatment guided by medication history. In regions with poorer economic conditions, individualized treatment guided by personal medication history could be considered as an alternative to antibiotic resistance testing. Additionally, more accurate, rapid, and patient-compliant detection methods need to be developed. The introduction of nanomaterials in recent years has made Hp diagnosis and treatment more precise and efficient, and this may represent another significant opportunity in the exploration of antibiotic resistance detection methods (Figure 1).
